# Clinical translation of surgical simulated closure of a ventricular septum defect

**DOI:** 10.1093/icvts/ivac122

**Published:** 2022-05-23

**Authors:** Qi Li, Nabil Hussein, Yunyi Zhang, Yibing Fang, Yue Wang, Qi An, Osami Honjo, Shuhua Luo

**Affiliations:** 1 Department of Anesthesiology, West China Hospital of Sichuan University, Chengdu, China; 2 Department of Congenital Cardiac Surgery, Yorkshire Heart Centre, Leeds General Infirmary, England, UK; 3 Department of Cardiovascular Surgery, Southwest Hospital of the Third Military Medical University, Chongqing, China; 4 Department of Cardiovascular Surgery, West China Hospital of Sichuan University, Chengdu, China; 5 Division of Cardiovascular Surgery, The Labatt Family Heart Centre, The Hospital for Sick Children, Toronto, ON, Canada; 6 Department of Surgery, University of Toronto, Toronto, ON, Canada

**Keywords:** Simulation, Congenital heart surgery, Ventricular septum defect, Education

## Abstract

**OBJECTIVES:**

To demonstrate that improvement in technical performance of congenital heart surgical trainees during ventricular septum defect (VSD) closure simulation translates to better patient outcomes.

**METHODS:**

Seven trainees were divided into 2 groups. Experienced-fellows group included 4 senior trainees who had performed >5 VSD closures. Residents group consisted of 3 residents who had never performed a VSD closure. Experienced-fellows completed 3 VSD closures on real patients as a pretest. Both groups participated in a 4-week simulation requiring each participant to complete 2 VSD closures on three-dimensional printed models per week. One month later, all trainees returned for a post-test operation in real patients. All performances were recorded, blinded and scored independently by 2 cardiac surgeons using the validated Hands-On Surgical Training–Congenital Heart Surgery (HOST-CHS). Predefined surgical outcomes were analysed.

**RESULTS:**

The median HOST-CHS score increased significantly from week 1 to 4 [50 (39, 58) vs 73 (65, 74), *P* < 0.001] during simulation. The improvement in the simulation of experienced-fellows successfully transferred to skill acquisition [HOST-CHS score 72.5 (71, 74) vs 54 (51, 60), *P* < 0.001], with better patients outcomes including shorter total cross-clamp time [pretest: 86 (70, 99) vs post-test: 60 (53, 64) min, *P* = 0.006] and reduced incidence of major patch leak requiring multiple pump runs [pretest: 4/11 vs post-test: 0/9, *P* = 0.043]. After simulation, the technical performance and surgical outcomes of Residents were comparable to Experienced-fellows in real patients, except for significantly longer cross-clamp time [Residents: 76.5 (71.7, 86.8) vs Experienced-fellows: 60 (53, 64) min, *P* = 0.002].

**CONCLUSIONS:**

Deliberate practice using simulation translates to better performance and surgical outcomes in real patients. Residents who had never completed a VSD closure could perform the procedures just as safely and effectively as their senior colleagues following simulation.

**Figure:**
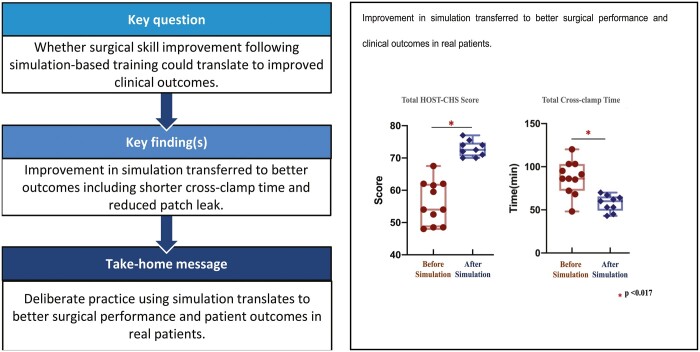

## INTRODUCTION

Congenital heart surgery (CHS) is one of the most technically demanding surgical specialties [1]. The current training of congenital heart surgeons relies largely on the traditional apprenticeship model (observation, assistance and supervised practice) and requires a long training period. This is primarily due to the wide variation and complexity of cardiac morphology, the limited exposure to rare lesion and the small size of patients [2]. Current survey data demonstrated that the mean duration of postgraduate training of congenital heart surgeons in North America was 10 years [3]. Surgical simulation is becoming an increasingly important educational tool in training adult cardiac surgeons, however, has yet to be established in CHS due to the paucity of effective simulation models [4–13]. Although recent studies have demonstrated the feasibility of three-dimensional (3D) printed models being used effectively in a variety of CHS simulation, the question remains whether these technical skill improvements translate to an improvement in clinical performance [14–17].

Perimembranous ventricular septum defect (VSD) closure is a principal component of 15% of all congenital heart operations [18]. Acquisition of skills related to VSD closure is an important step in the development of congenital heart surgeons. According to the American Board of Thoracic Surgery, VSD closure was one of the specific qualifying major procedures of the operative requirements for CHS sub-specialty certification [19]. There has been a recent effort to develop and validate simulation methods in CHS using objective, procedure-specific assessment tools [20]; however, translation to clinical practice is lacking. Taking advantage of this new platform, we aimed to quantify whether surgical skill improvement following simulation-based training on 3D-printed VSD models could translate to improved clinical outcomes.

## MATERIALS AND METHODS

A total of 7 trainees of varying levels of experience in CHS participated in our VSD simulation curriculum and were divided into 2 groups according to their previous experience in CHS **(**Supplementary Material, Table SE1). Experienced-fellows group included 4 trainees (2 junior staff and 2 fellows) who had performed at least 5 VSD closure as the primary surgeon. Residents group consisted of 3 residents who had never performed a VSD closure.

### Ethical statement

The study was registered on the Chinese Clinical Trial Registry (ChiCTR2000033192, 23 May 2020). Consent of patients was confirmed before surgery. Approval was obtained from the Research Ethics Board at West China Hospital for filming and analysing the performance of participants, all of whom consented to the study [2020 (386), 11 May 2020].

### Three-dimensional printed models

**Figure 1:**
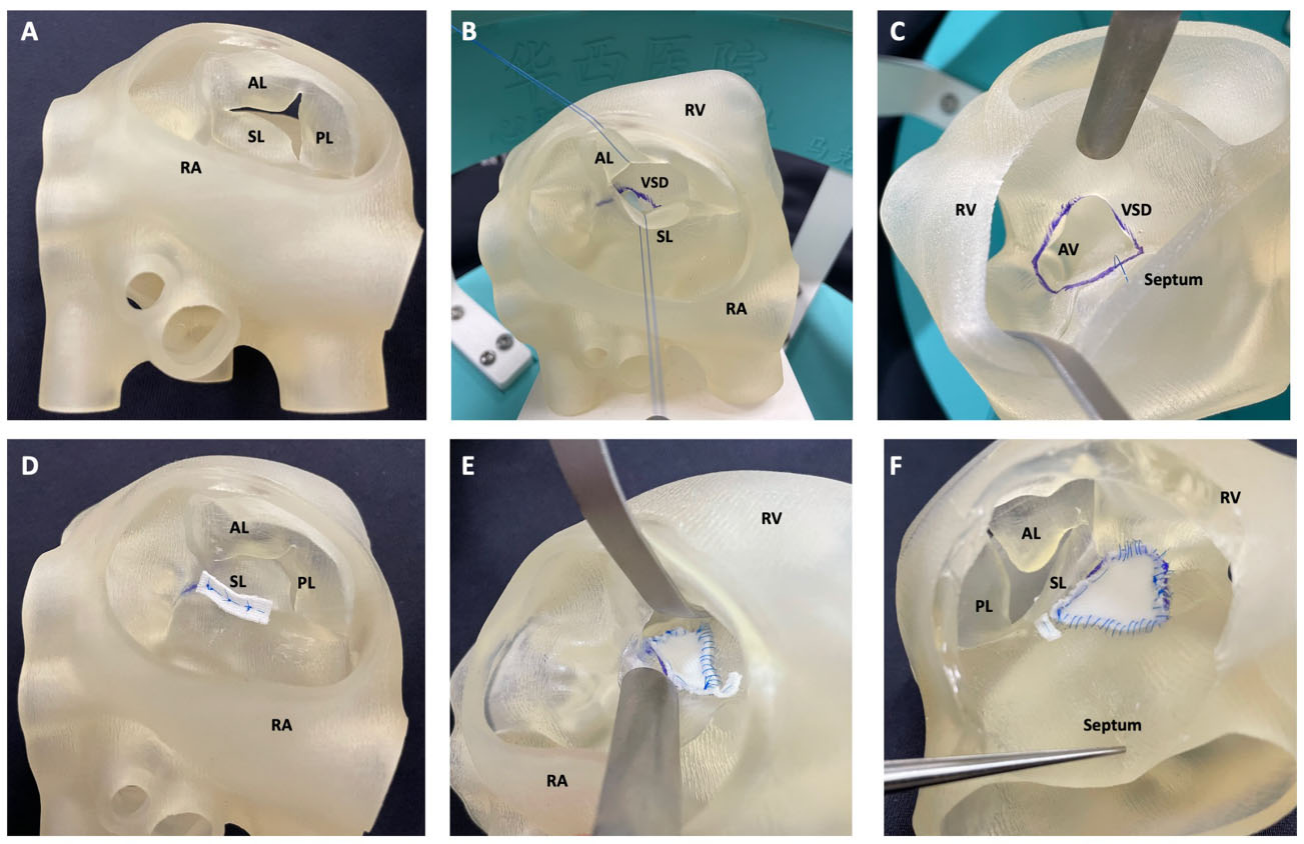
Three-dimensional printed Heart model before and after simulation. The views (via right atrium: **A**, **B**, **D**, and **E**; via right ventricle: **C**, and **F**) of a three-dimensional-printed heart model before (**A–C**) and after (**D–F**) simulation. AL: anterior leaflet; AV: aortic valve; PL: posterior leaflet; RA: right atrium; RV: right ventricle; SL: septal leaflet; VSD: ventricular septum defect.

The models were 3D printed as previously described [17] (Fig. 1). A contrast-enhanced computed tomography scan was obtained from an 8-month old, 9 kg patient with an unrestrictive perimembranous VSD to develop the model. The original Digital Imaging and Communications in Medicine image data were postprocessed using commercially available software programs (Mimics^®^ 21.0 and 3-Matic^®^13.0, Materialise, Leuven, Belgium).

The volume-rendered Digital Imaging and Communications in Medicine image files produced were converted to the Standard Tessellation Language files for computer-aided design and printing (Video 1). The tricuspid valve leaflets were segmented from the original images. The model was then placed on a graphically designed platform so that it could be secured to a table, with supporting stools to keep it in the anatomical position. Three-dimensional printing was performed on a commercially available 3D printer (J750 Digital Anatomy printer, Stratasys Ltd, Minnesota, USA) using the most flexible material (GelMatrix resin, TissueMatix resin and Agilus 30 clear) for the heart, a solid material (VeroWhite) for the platform and stools, and mixture of the 2 print materials for valvar annuli. The leaflets were printed with a flexible-transparent material (UV Curable Resin, FLX910T). Printing time was 15–18 h for each model.

### Curriculum

**Figure 2:**
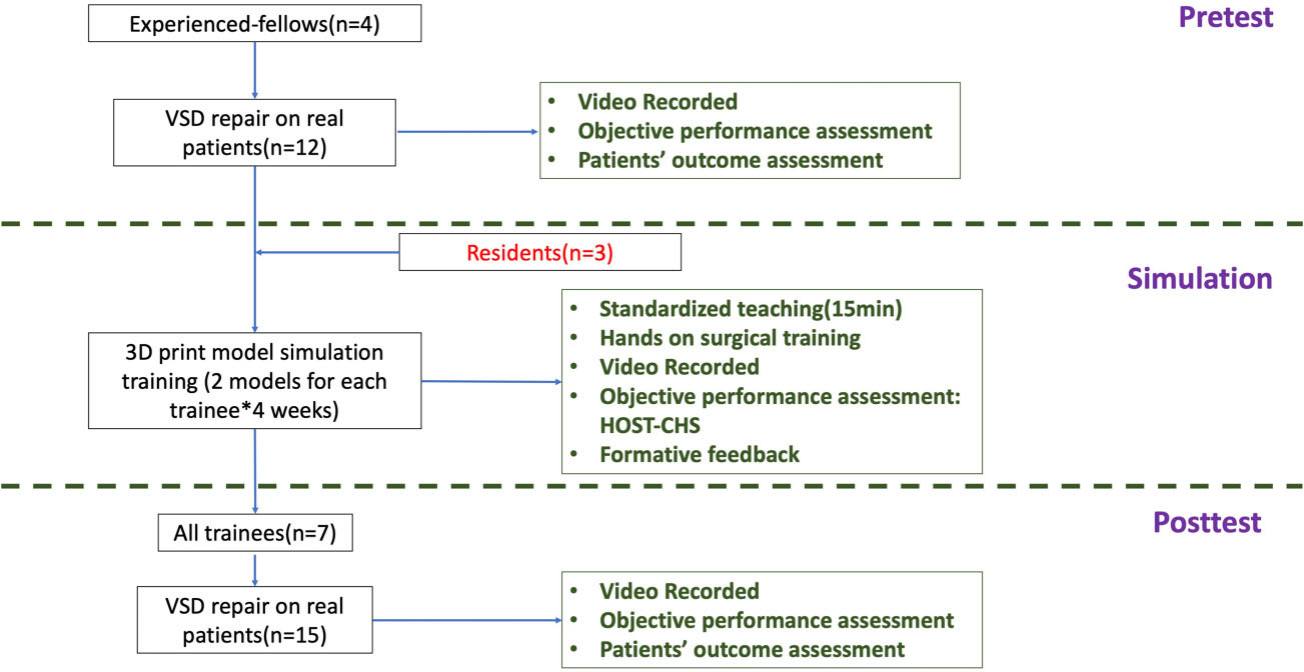
Surgical simulation curriculum for ventricular septum defect closure. A total of 7 trainees participated in the ventricular septum defect simulation curriculum and were divided into 2 groups. Experienced-fellows group included 4 trainees (2 junior staff and 2 fellows) who have performed at least 5 VSD closure as primary surgeon. Residents group consisted of 3 residents who had never performed a VSD closure. The curriculum commenced with a pretest requiring trainees from Experienced-fellows group to perform VSD closures in the operating room. Both groups participated in a 4-week simulation programme requiring each participant to complete 2 VSD closures on three-dimensional printed models weekly. After a 1-month delay, all trainees returned for a post-test in real patients and were re-evaluated. Pretest, simulation and post-test performances were filmed, and graded blindly and independently by 2 cardiac surgeons using Hands-On Surgical Training–Congenital Heart Surgery (HOST). The predefined surgical outcomes of patients were recorded. HOST: Hands-On Surgical Training–Congenital Heart Surgery; VSD: ventricular septum defect.

The curriculum commenced with a pretest requiring trainees from Experienced-fellows group to perform VSD closures in the operating room (3 patients per trainee) (Fig. 2). The procedure was supervised by senior cardiac surgeons and was video recorded for retrospective objective assessment. If operations were deemed to be going on for too long (cross-clamp time > 90 min) or a requirement for second pump run, the supervising surgeon would take over and complete the case. The trainee’s actions had to be corrected by the supervising surgeon at some point during the operation. Typical reasons that this scenario would take place were (1) deep suture bites being placed in the region of the His Bundle that could potentially injure the conducting system; (2) inaccurate suture bites that may catch the aortic valve; and (3) if sutures were a risk of tethering or rupturing the tricuspid valve leaflets.

Intraoperative transoesophageal echocardiography (TEE) was routinely performed to document the adequacy of the closure. Residual lesions requiring a second pump run included: (1) patch leak with pulmonary to systemic flow ratio (Qp:Qs) greater than 1.5 (calculated by saturation of blood from the right atrium and pulmonary artery) and (2) any postoperative new-onset or increased tricuspid or aortic valve regurgitation.

These trainees were then entered into a 4-week simulation-based training programme requiring each trainee to complete 2 VSD closures on 3D-printed models each week. Residents group (*n* = 3) was added to this programme who had not previously performed VSD closure on real patients. Instruments, sutures and patches were provided. A standardized 15-min lecture and a video demonstrating the important steps of VSD closure were created by a senior paediatric cardiac surgeon and given to trainees prior to the simulation programme. Formative feedback was provided after each practice by the supervising surgeons while discussing results such as the shape of the patch, suture placement, residual VSD and collateral damage. After a 1-month delay (including no exposure to any VSD-related surgeries), all trainees (*n* = 7) returned for a post-test with each trainees performing at least 2 VSD closures on real patients.

Performance on the pretest, simulations and post-test was recorded. Each video was stripped of identifying information and then assessed independently by 2 cardiac surgeons (YF and SL) who were blinded to participant identity and the type of test (i.e. pretest versus post-test). Patients who were diagnosed with a simple unrestrictive VSD and had similar body weight and age to the sample patient used to generate the 3D-printed model were selected for the pre- and post-tests. A survey was conducted on trainees 1 month after the post-test to evaluate the feedback of the simulation.

### Performance assessment

The Hands-On Surgical Training–Congenital Heart Surgery (HOST-CHS), a previously validated procedure-specific assessment tool, was used to objectively assess technical skills performance in both simulation and the operating room [20] (Supplementary Material, Table SE2). The VSD closure was broken down into 21 tasks under 4 broad sections: (1)preparation of VSD patch; (2)suturing of VSD patch; (3)patch assessment; and (4)collateral damage. A binary method of assessment was used for each step which was weighted based on its overall importance in the operation using a Likert scale of 1 to 5 (5 signifying highest importance and 1 representing lowest importance), with a maximal HOST-CHS score of 77. In order to further incorporate the general aspects of the VSD closure operation, each question of the HOST-CHS assessment tool was evaluated and placed into 1 of 3 categories: (1) fluency of the procedure (e.g., suture assessment); (2) knowledge of the technical aspects of the procedure (e.g. preparation of VSD patch); and (3) respect for tissue/patch (e.g. any visible holes/tears within the patch, avoidance of collateral damage). Time from the preparation of the VSD patch to the tying of the final knot was also recorded.

### Operative technique

The surgical technique of perimembranous VSD closure was standardized in our centre. Cardiopulmonary bypass was established with mild hypothermia (32°C–34°C) by standard aortic and bicaval venous cannulation. Myocardial protection was achieved with antegrade cold blood cardioplegia. The VSD and the important surrounding structures were exposed through the transatrial approach. The size and shape of VSD were measured (step 1) and the Dacron patch was trimmed to the approximate size accordingly (step 2). A running 6-0 polypropylene sutures with a BV1 needle (Ethicon, Inc, Somerville, NJ, USA) was usually used for VSD closure starting at the deepest point along the ventricular crest (point A, step 3; Supplementary Material, Fig. SE1). The suture continued superiorly towards the aortic annulus (side 1, step 4), and around the aortic valve towards the tricuspid valve annulus (side 2, step 5). Care was taken not to catch any aortic valve cusps (step 6). When the tricuspid annulus was reached (point B), the suture was passed through the annulus in the right atrium (step 7). The other end of the suture then continued along the inferior margin of the VSD (side 3) to the tricuspid valve annulus (point C, step 8) after trimming the patch along the inferior rim of VSD if required (step 10). The suture bites stayed well to the right side of the septal crest and were relatively superficial to avoid conduction tissue injury (step 9) when reached the area where the His bundle lies. Care was taken to avoid tethering/rupturing the tricuspid valve chords (step 11). The suture was passed through the annulus in the right atrium when the tricuspid annulus was reached with or without a transitional stitch (step 12). The repair was completed with several horizontal mattress sutures with pericardium pledgets. Septal leaflet or chordal detachment is not our routine for hard-to-expose VSDs. If the margins of the VSD were deemed crowded and hidden from view by tight chordal attachment, or the presence of multiple chordae, the secondary chords attached to the ventricular surface in the body of the tricuspid leaflets were usually divided to facilitate VSD exposure. Any chords division on pretest and post-test was recorded.

### Data collection

Patients’ demographic data, perioperative variables, intraoperative information including the results of intraoperative TEE and postoperative data were collected. The primary outcomes were patient outcomes including the cross-clamp time, requirement of a second pump run, haemodynamically significant lesions shown by intraoperative TEE (patch leak, any postoperative new-onset or increased tricuspid or aortic valve regurgitation) and atrioventricular block requiring a pacemaker. The secondary outcome was the performance in the operating room assessed by HOST-CHS and time-to-completion. Other outcomes evaluated included length of intensive care unit stay, duration of ventilation and hepatic and renal failure.

### Statistical analysis

Descriptive statistics for categorical variables are reported as frequency and percentage. Continuous variables were expressed as a median and interquartile range and categorical variables as absolute numbers with percentages. Categorical variables were compared between groups using the Fisher’s exact test, and continuous variables were compared using analysis of variance or the Wilcoxon rank-sum test, where appropriate. Variables comparison within Experienced-fellows group was analysed with paired *t*-test or the Wilcoxon test, where appropriate. An adjusting *P*-value of 0.017 was considered as the significance level in multiple testing. Internal consistency was calculated using Cronbach’s alpha. Interrater reliability was calculated using intraclass correlation coefficients. All statistical tests were 2-sided with the alpha level set at 0.05 for statistical significance. Statistical analysis was performed with Stata version 14.0.

## RESULTS

### Pretest technical performance and surgical outcomes

A total of 11 VSD closures were completed in the pretest by 4 trainees in Experienced-fellows group. The median age and weight of these patients were 12 (8, 17) months and 9.5 (6.65, 12) kg, respectively. One trainee (T2) was taken over by the supervising surgeon in his third pretest case due to a long cross-clamp time.

**Figure 3:**
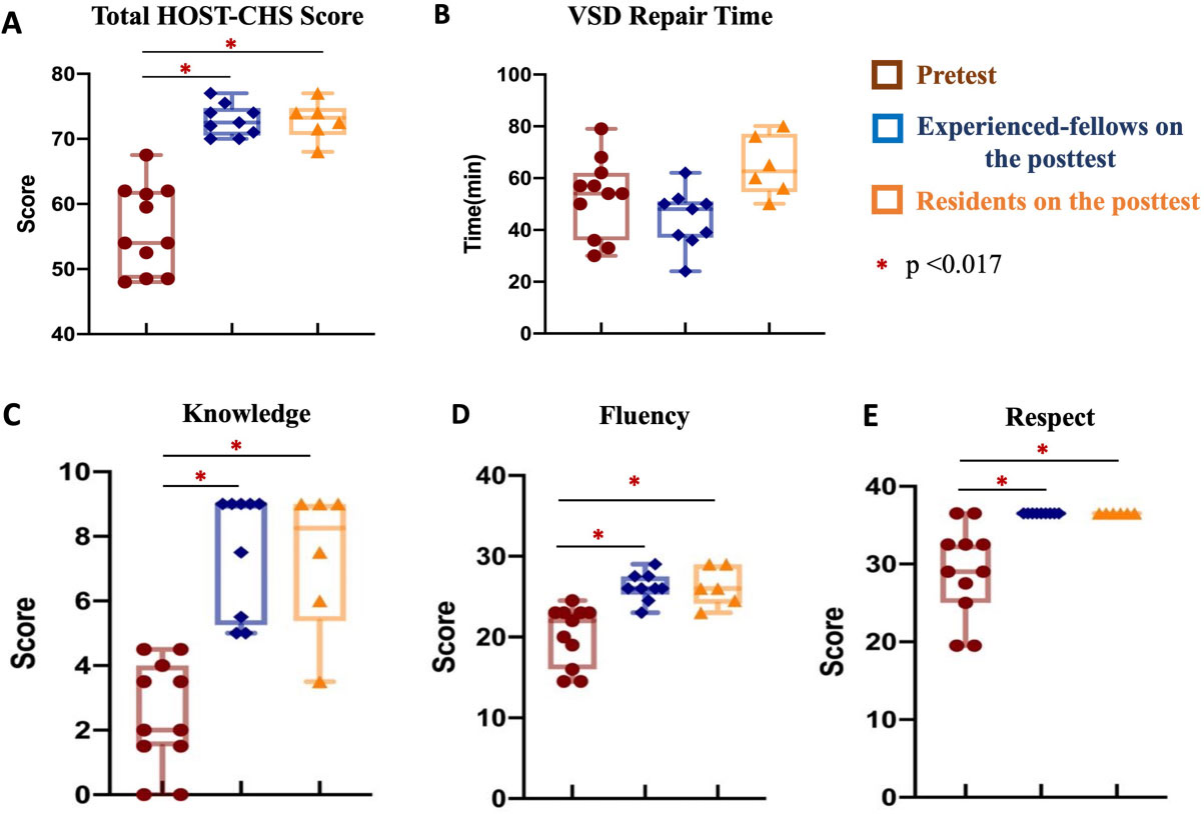
The comparison of technical performance on pretest and post-test. Pretest results and post-test results for Experienced-fellows and Residents are shown for (**A**) VSD closure time, (**B**) total HOST-CHS score and the 3 holistic HOST-CHS categories of (**C**) Knowledge, (**D**) Fluency and (**E**) Respect. The horizontal line in the box plot indicates the mean and the box indicates the upper and lower quartiles, with the vertical lines representing the minimum and maximum values. HOST-CHS: Hands-On Surgical Training–Congenital Heart Surgery; VSD: ventricular septum defect.

**Figure 4:**
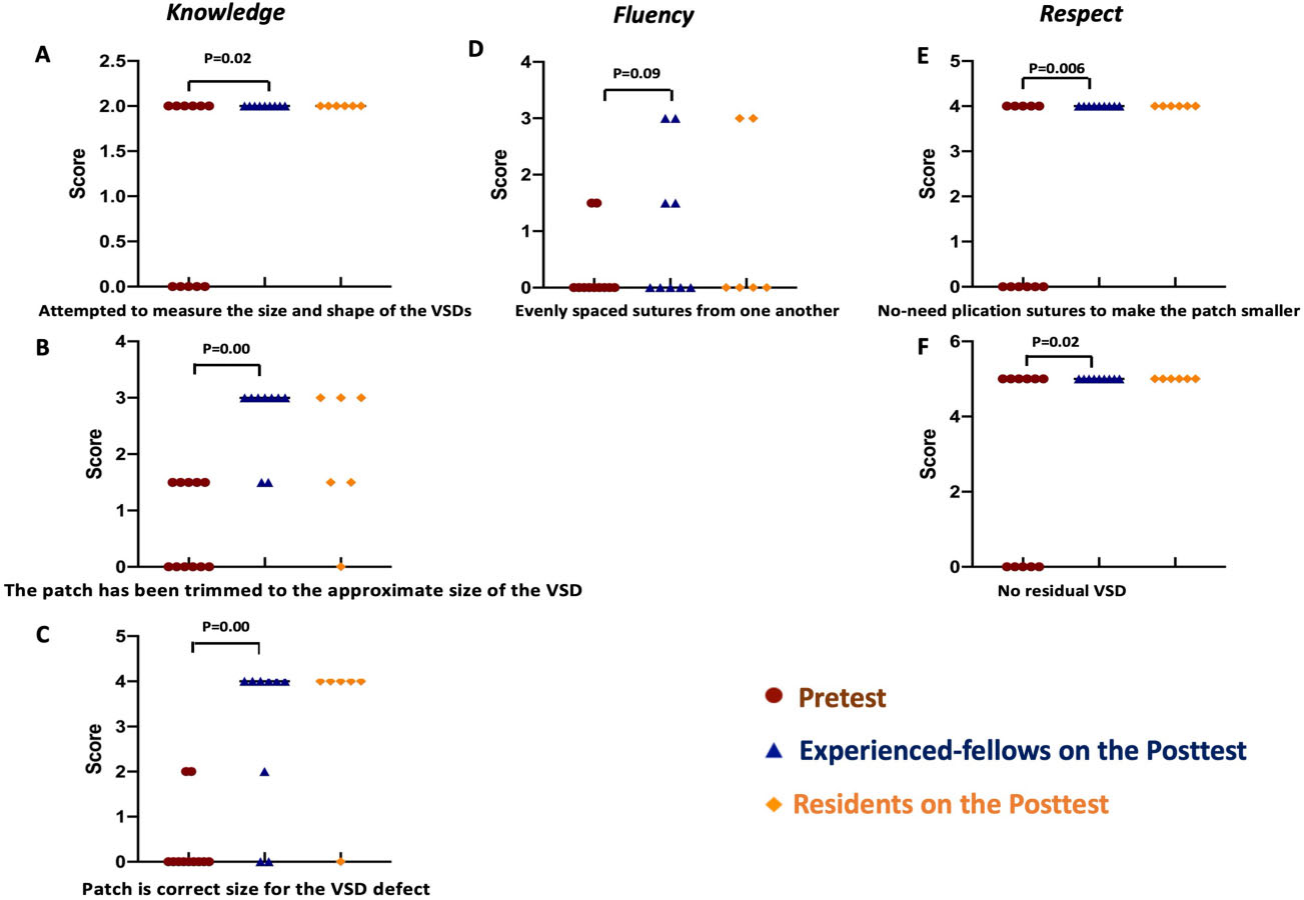
Deficient technical tasks on the pretest and post-test. Each dot represents the mean score of technical tasks in individual patient. Pretest results and post-test results for Experienced-fellows and Residents are shown for Knowledge tasks of (**A**) measuring the size and shape of the VSDs, (**B**) trimming patch to approximate size of VSD, (**C**) appropriateness of patch size; Fluency tasks of (**D**) even suture placement; Respect tasks of (**E**) avoidance of plication sutures and (**F**) absence of residual VSD. VSD: ventricular septum defect.

The technical performance of each trainee on the pretest was shown in Supplementary Material, Table SE3. The median total HOST-CHS score was 54 (51, 60). For the holistic HOST-CHS scores the trainees scored lowest overall for knowledge of the technical aspects of the procedure [2.5 (0, 4.5)/9], followed by fluency of the procedure [20.2 (14.5, 24.5)/29] and respect for tissue/patch [35 (29, 39)/39] (Fig. 3)**.** The most common deficient technical tasks related to knowledge included attempt to measure the size and shape of the VSD [1.1 (0–2)/2], appropriate initial patch-trimming [0.82 (0–1.5)/3] and correct size of final patch [0.55 (0–2)/4] (Fig. 4). Suture placement [0.41 (0–1.5)/3] had the lowest score in the tasks within the fluency category whereas the use of plication sutures [1.82 (0–4)/5] and the likelihood of a residual VSD [2.72 (0–5)/5] were the most 2 common technical deficiencies among the tasks related to respect. The median VSD repair duration was 54 (36, 62) min.

**Figure 5:**
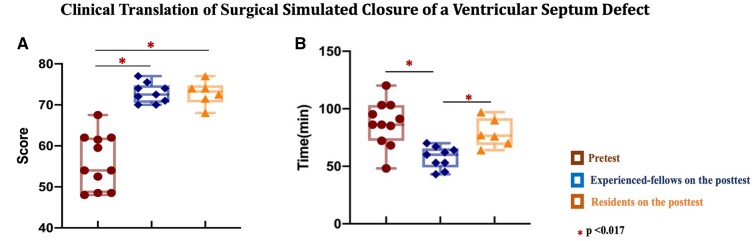
Graphical display of the translatability of improvement in simulation to surgical performance of congenital heart surgical trainees and outcomes of patients undergoing ventricular septum defect closure. *Study design:* A prospective single-centre study. *Methods:* Experienced-fellows completed 3 VSD closures on real patients as a pretest. Both Experienced-fellows (*n* = 4) and Residents (*n* = 3) participated in a 4-week simulation. All trainees returned for a post-test operation in real patients (*n* = 15). *Results:* The improvement in the simulation of Experienced-fellows successfully transferred to skill acquisition (**A**), shorter total cross-clamp time (**B**) and reduced major patch leak (*P* = 0.043) in real patients. After simulation, the technical performance of Residents was comparable to Experienced-fellows despite longer cross-clamp time. *Implication:* Improvement in simulation translates to better performance and surgical outcomes in patients. HOST-CHS: Hands-On Surgical Training–Congenital Heart Surgery; VSD: ventricular septum defect.

The total cross-clamp time was 86 (70, 99) min, with more than one-third of patients (*n* = 4, 36.4%) requiring a second pump run due to a significant patch leak (Supplementary Material, Table SE4). There was no moderate or severe tricuspid or aortic valve regurgitation on the intraoperative TEE following the repair. Despite the frequent requirement of multiple pump runs, patients had an uneventful intensive care unit recovery, with a median ventilation duration of 11 (5, 28.5) h and a median intensive care unit duration of 3 (3, 6.5) days. No patients had atrioventricular block requiring pacemaker, hepatic failure or renal failure.

### Simulation training using three-dimensional printed models

The total HOST-CHS score of all 7 trainees gradually increased throughout the training period, while the time-to-completion remained unchanged (Supplementary Material, Fig. SE2). The HOST-CHS score and the time-to-completion at each week were comparable between 2 groups of trainees. Simulation significantly improved holistic scores of all 3 aspects including knowledge [Week 1: 5 (0–9) vs Week 4: 9 (5–9), *P* < 0.001], fluency [Week 1: 26 (16–29) vs Week 4:27.5 (22–29), *P* = 0.04] and respect [Week 1: 22 (10–34) vs Week 4: 35 (25–39), *P* < 0.001] (Supplementary Material, Fig. SE3). The majority of low scoring tasks on the pretest had significantly improved following simulation training; however, the score of suture placement (*P* = 1.00) and need of patch plication suture (*P* = 0.46) remained the same (Supplementary Material, Fig. SE4).

### Post-test technical performance and surgical outcomes

A total of 15 VSD closures were completed by 7 trainees as primary surgeon in the operating room (9 in Experienced-fellows group, 6 in Residents group). No case was taken over by the supervising surgeon in the post-test. The median age and weight of patients were 11 (8.5, 15) months and 9 (6.5, 13.5) kg, respectively (Supplementary Material, Table SE5).

The technical performance of each trainee in the post-test is shown in Supplementary Material, Table SE3. The post-test total HOST-CHS score [72.5 (71, 74) vs 54 (51, 60), *P* < 0.001] and holistic scores of all 3 categories [knowledge: 9 (5.5, 9) vs 2.5 (0, 4.5), *P* < 0.001; fluency: 26 (26, 27.5) vs 20.2 (14.5, 24.5), *P* = 0.001; respect: 39 (39, 39) vs 35 (29, 39), *P* = 0.003] in Experienced-fellows group were significantly higher than the pretest scores. Additionally, the VSD closure duration was shorter on post-test compared to the pretest, although the difference was not significant [48 (38, 50) vs 54 (36, 62) min, *P* = 0.18] (Fig. 3)*.* The main deficiency on the post-test was in suture placement only [1 (0–3)/3] (Fig. 4). The surgical outcomes of Experienced-fellows group on the post-test were better than the pretest, including significantly shorter total cross-clamp times [pretest: 86 (70, 99) vs post-test: 60 (53, 64) min, *P* = 0.006] and reduction in major patch leak requiring multiple pump runs [pretest: 4/11 vs post-test: 0/9, *P* = 0.043] (Table 1).

**Table 1:** Patient outcomes on the pretest and post-test

Parameters	Pretest	Post-test		
		Post-test (sum)	Post-test case (Experienced-fellows group)	Post-test case (Residents group)
Clamp time for first pump run (min)	70 (50.3, 83)	64 (56.5, 73)	60 (53, 64)	76.5 (71.7, 86.8)
Total cross-clamp time (min)	86 (70, 99)	64 (56.5, 73)	60 (53, 64)^a^	76.5 (71.7, 86.8)^b^
Multiple pump run (*n*)	4/11	0/15	0/9	0/6
Patch leak (*n*)	4/11	1/15	1/9	0/6
Important patch leak (*n*)	4/11ggrou	0/15	0/9	0/6
Postoperative new-onset or increased TR	0/11	0/15	0/9	0/6
Postoperative new-onset or increased AR	0/11	0/15	0/9	0/6
ICU duration (day)	3 (3, 6.5)	4 (3, 4.8)	4 (3, 4)	4 (3, 5)
Ventilation duration (h)	11 (5, 28.5)	6 (4, 10.5)	7 (5, 11)	4 (2, 4)
AV block required pacemaker	0/11	0/15	0/9	0/6
Liver failure	0/11	1/15	1/9	0/6
Renal failure	0/11	0/15	0/9	0/6

a Between post-test cases and pretest cases performed by Experienced-fellows group, *P* = 0.006.

b Between post-test cases performed by Experienced-fellows group and post-test cases performed by Residents group, *P* = 0.002.

AR: aortic valve regurgitation; AV: atrioventricular; ICU: intensive care unit; TR: tricuspid valve regurgitation.

Trainees in Residents group performed the VSD closure with a significantly higher total HOST-CHS score [73.3 (71.5, 74) vs 54 (51, 60), *P* < 0.001] and holistic scores [knowledge: 8.3 (6, 9) vs 2.5 (0, 4.5), *P* = 0.001; fluency: 26 (26, 29) vs 20.2 (14.5, 24.5), *P* < 0.001; respect: 39 (39, 39) vs 35 (29, 39), *P* = 0.013] than Experienced-fellows group on the pretest, within comparable VSD closure duration [Resident group: 62.5 (56, 76) vs Experienced-fellows group: 54 (36, 62) min *P* = 0.14] and total cross-clamp time [Resident group: 76.5 (71.7, 86.8) vs Experienced-fellows group: 86 (70, 99) min, *P* = 0.45] (Fig. 3 and Table 1).

The surgical performance of Residents group was similar to Experienced-fellows group on the post-test [total HOST-CHS, 73.3 (71.5, 74) vs.72.5 (71, 74), *P* = 1.0; knowledge: 8.3 (6, 9) vs 9 (5.5, 9), *P* = 0.84; fluency: 26 (26, 29) vs. 26 (26, 29), *P* = 0.94; respect: 39 (39, 39) vs 39 (39, 39), *P* = 1.0]; however, the VSD closure duration was significantly longer in Residents group [Residents group: 62.5 (56, 76) vs Experienced-fellows group: 48 (38, 50) min, *P* = 0.005]. The primary outcomes in Residents group were comparable to Experienced-fellows group on the post-test, except for significantly longer cross-clamp time [Residents group: 76.5 (71.7, 86.8) vs Experienced-fellows group 60 (53, 64) min, *P* = 0.002]. There was no significant difference in postoperative recovery with a comparable result between the 2 groups of trainees on the post-test (Table 1).

A total of 26 videos (pretest = 11, post-test = 15) were independently assessed by 2 cardiac surgeons. The interrater and intrarater reliabilities of HOST-CHS assessment tool were α = 0.8021 and ICC = 0.944, respectively, demonstrating a high level of consistency.

As shown in Supplementary Material, Table SE6, all trainees agreed that the simulation improved their surgical skills throughout all the domains including VSD patch preparation, suturing, assessment and the avoidance of collateral damage.

## DISCUSSION

The current study was undertaken to demonstrate surgical simulation’s effectiveness in teaching VSD closure, the most commonly performed operation in CHS. This analysis is unique when compared to previously published reports [4–13]. The results showed that early simulation training significantly improved trainees’ performance in the operating room, which corresponded into better surgical outcomes. After dedicated simulation-based training, residents who have never done a VSD closure could perform the operation as well as their seniors who have intraoperative experience in VSD closure as the primary surgeon. These findings provided objective and quantitative evidence of improved technical performance and highlighted its translatability to improved intraoperative and postoperative outcomes [21] (Fig. 5).

### Paradox of traditional training model

The training of congenital heart surgeons is extremely complex and challenging [19, 22, 23]. American Board of Thoracic Surgery currently requires fellows to complete a minimum of 5 VSDs as the primary surgeon to achieve CHS sub-specialization certification [19]. However, 5 VSD closures may not be enough for adequate skills acquisition under the traditional apprenticeship model. According to the current study, trainees who had performed up to 10–20 VSD closures still demonstrate some technical deficiencies throughout all steps of VSD closures in real patients. Expectedly, the surgical outcomes on the pretest were suboptimal with more than one-third of cases requiring a second pump run for significant patch leak. Our results highlighted the paradox of the current traditional training model whereby surgical exposure as the primary surgeon is crucial to achieve a certain level of proficiency, however, this is challenging to achieve due to the growing expectation of perfect outcomes and ongoing surgeon scrutiny.

### Skills acquisition in simulated ventricular septal defect closure

This simulation model enabled dedicated practice by providing a low-risk, inconsequential environment for surgeons to hone their skill in order to perform better in reality [24]. Using the benefits of expert surgeons’ experience, a ‘successful VSD closure’ was divided into 21 tasks with a well-defined goal in the HOST-CHS assessment tool. Trainees were able to refine their performance based on immediate structured feedback, including an objective score that highlighted the specific areas and tasks that needed improving. During simulation, certain competencies related to fluency or respect for tissue were longer to acquire than knowledge of the procedure. This may suggest that these general skills take longer to develop/achieve competency in. Identification of such tasks is useful in the future design and delivery of simulation-based education.

### Improvement of technique performance in operating room and patient outcomes

While simulation is perceived to be favourable in cardiac surgery, authors have struggled to demonstrate the merit of Kirkpatrick’s higher level of outcomes evaluation [21]. The current study observed a significant improvement in technical performance and surgical outcomes after simulation, highlighting the importance of simulation as a powerful surgical training tool in CHS. Although the VSD closure duration was not shortened, the total cross-clamp time on the post-test was significantly reduced. In addition to improved surgical efficiency, there was significantly reduction in the incidence of residual patch leak with no post-test patients having atrioventricular block or significant tricuspid valve regurgitation.

Residents achieved comparable technical performance and surgical outcomes to the relatively experienced fellows/junior staff following simulation. Furthermore, the technical performance of residents after simulation was significantly better than their seniors before simulation. Consistent with Hoashi *et al.* [15], these results supported the belief that effective simulation can be used to prepare inexperienced surgeons to perform the CHS cases with favourable performance and outcomes. This is particularly attractive in the training of congenital heart surgeons as the long training period has become one of the major concerns under current patient-based training models. By incorporating the HOST curriculum in CHS training programmes may help augment traditional patient-based training, especially for complex and rare heart lesions. Further studies are warranted to compare the outcomes between inexperienced surgeons with/without simulator exposure in surgical management of complex heart lesions.

### Limitations and future directions

The major limitations of this study include multiple confounding variable in assessing the operating performance of the trainees such as exposure of VSDs and nature of VSDs were not controlled due to the small number of trainees limiting the generalizability of the results. Another limitation was the significant variability in experience of trainees even within one group (Experienced-fellows vs Residents). Therefore, the paper should be positioned as a hypothesis-generating pilot study. In real practice, avoidance of sutures tangling with or tethering the chordae is important technical considerations. Unfortunately, this could not be reflected and assessed in simulation-based training as the tricuspid chordae tendineae was not printed in our 3D printed model due to the inadequate image quality. However, we propose that technical skills such as patch management, needle spacing and avoidance of collateral damages must be mastered before any attempts are made at VSD closure in the clinical setting.

Our ultimate goal is to incorporate HOST into current CHS training programmes, in order to offer current and future trainee surgeons a robust pathway to success by providing simulation-based standardized education without compromising patient care. To achieve this there are several refinements left to do:

1. Improvement of model material: The current models are more difficult to suture and can easily tear when compared to real myocardium and endocardium. Ongoing collaboration between materials scientists and 3D-printed material manufacturers will undoubtedly expedite the pace of rapid advances within this field.
2. Addition of subvalvular apparatus to models to better simulate reality: The continuing advancement of 3D printing technologies, and the emergence of a variety of digital materials and material composites, will allow more accurate anatomic replication of tricuspid valve apparatus shortly.
3. Study of more complex CHS procedures: Learning of neonatal complex cardiac surgery poses the greatest challenge in CHS training. Future studies can consider establishing learning objectives for lesions including total pulmonary vein anomalous drainage, transpositions or hypoplastic left heart syndrome.

Encouraged by the current result, we recently launched a programme in China, trying to incorporate 3D-printing model-based surgical simulations into the training of congenital heart surgical residents and fellows from multiple Chinese paediatric heart centres.

## CONCLUSION

This study demonstrated that deliberate practice using simulation translates to better performance and surgical outcomes in real patients. Furthermore, residents who have never completed a VSD closure could perform the procedures just as safely and effectively as their senior colleagues, following simulation. The incorporation of curriculums consisting of high-fidelity simulators and tailored objective assessment methods into CHS training programmes should be considered for the next generation of congenital heart surgeons.

## SUPPLEMENTARY MATERIAL

Supplementary material is available at *ICVTS* online.

**Conflict of interest:** none declared.

## Supplementary Material

ivac122_Supplementary_Data

## Data Availability

The data underlying this article will be shared on reasonable request to the corresponding author.

## Abbreviations

3D: Three-dimensional
CHS: Congenital heart surgery
HOST-CHS: Hands-On Surgical Training–Congenital Heart Surgery
TEE: Transoesophageal echocardiography
VSD: Ventricular septum defect
