# Supplementary material for: Clinical translation of surgical simulated closure of a ventricular septum defect
Source: Interact Cardiovasc Thorac Surg. 2022 May 23;35(3):ivac122. doi: 10.1093/icvts/ivac122 (PMC9486874; doi:10.1093/icvts/ivac122)
Supplement: ivac122_Supplementary_Data [file ivac122_supplementary_data.zip › Supplemental Figure Legends.docx]

**Supplemental Figure Legends**

**Figure E1: Standardized Surgical Techniques of Preimembranous Ventricular Septum Defect Closure**

**Figure E2: Skill Acquisition Throughout Simulation Training using 3D-printed Models** Progression of total HOST-CHS score and duration of VSD closure throughout 4-week simulation curriculum as analyzed overall and as subgroup analyses for Experienced-fellows and Residents (C, D). The horizontal line in the box plot indicates the mean and the box indicates the upper and lower quartiles, with the vertical lines representing the minimum and maximum values.

HOST-CHS: Hands-On Surgical Training–Congenital Heart Surgery; VSD: ventricular septum defect

**Figure E3: Improvement of Holistic Scores on 3D-pritned Models following Simulation** Simulation significantly improved holistic scores of all three aspects including knowledge(A), fluency(B) and respect(C) on 3D-printed models. The horizontal line in the box plot indicates the mean and the box indicates the upper and lower quartiles, with the vertical lines representing the minimum and maximum values.

**Figure E4: Deficient Technical Tasks Before and After Simulation**

Each dot represents the mean score of technical tasks in individual model. Results of week-one and week-four of simulation for all trainees are shown for Knowledge tasks of (A) measuring the size and shape of the VSDs, (B) trimming patch to approximate size of VSD, (C) appropriateness of patch size; Fluency tasks of (D) even suture placement; Respect tasks of (E) avoidance of plication sutures and (F) absence of residual VSD.

VSD: ventricle septum defect

**Supplemental Video 1: The Reconstructed 3D Image for Heart Model Printing**

Red line: rim of the ventricle septum defect; Yellow area: the tricuspid valve leaflets
