# Supplementary material for: Clinical translation of surgical simulated closure of a ventricular septum defect
Source: Interact Cardiovasc Thorac Surg. 2022 May 23;35(3):ivac122. doi: 10.1093/icvts/ivac122 (PMC9486874; doi:10.1093/icvts/ivac122)
Supplement: ivac122_Supplementary_Data [file ivac122_supplementary_data.zip › Supplementary materials without mark .docx]

**Supplemental Materials**

**Table E1: The VSD Surgical experience of Trainees before 3D HOST training**

| **Trainees** | **Level** | **Group** | **VSD closure as primary surgeon(n)** | **VSD repair as a first assistant(n)** | **Other simple CHD surgery as a primary surgeon(n)** | **Other simple CHD surgery as a first assistant(n)** |
| --- | --- | --- | --- | --- | --- | --- |
| T1 | Junior staff | Expirenced-fellows | 10-30 | >50 | >50 | >100 |
| T2 | Junior staff | Expirenced-fellows | 10-30 | >50 | >50 | >100 |
| T3 | Fellow | Expirenced-fellows | 5-10 | 30-50 | 30-50 | >50 |
| T4 | Fellow | Expirenced-fellows | 5-10 | >50 | >50 | >100 |
| T5 | Resident | Residents | 0 | >50 | 10-30 | >100 |
| T6 | Resident | Residents | 0 | 10-30 | 0-10 | 0-10 |
| T7 | Resident | Residents | 0 | 0 | 30-50 | >50 |

**Abbreviations:** VSD: ventricular septum defect; CHD: congenital heart disease

**Table E2: HOST-CHS Assessment Tool for VSD closure**

**Abbreviations:** VSD: ventricular septum defect; HOST-CHS: Hand On Surgical Training-Congenital Heart Surgery

**Table E3: Technique Performance of Trainees on the Pretest and Posttest**

| **Trainees** | **Group** | **Pretest** | | | | | **Posttest** | | | | | **P value^*^** | **P value^#^** |
| --- | --- | --- | --- | --- | --- | --- | --- | --- | --- | --- | --- | --- | --- |
|  |  | **HOST-CHS Score** | | | | **VSD closure time(min)** | **HOST-CHS Score** | | | | **VSD closure time(min)** |  |  |
|  |  | **Total** | **Knowledge** | **Fluency** | **Respect** |  | **Total** | **Knowledge** | **Fluency** | **Respect** |  |  |  |
| T1 | Experienced-fellows | 55(51.5,58.5) | 2(1,3.3) | 16(15.3,19.5) | 27.5(23.5,32) | 53.5(51.8,55.3) | 74(74,74) | 9(8.25,9) | 26(25.3,26.8) | 36.5(36.5,36.5) | 38(31,38.5) | 0.01 | 0.01 |
| T2 | Experienced-fellows | 62(61,63) | 3.8(3.6,3.9) | 23.8(23.4,24.1) | 34.5(33.5,35.5) | 49(42.5,55.5) | 72(69.5,74.5) | 7.3(6.4,8.1) | 28.3(27.9,28.6) | 36.5(36.5,36.5) | 56(53,59) | 0.02 | 0.08 |
| T3 | Experienced-fellows | 54(52.5,56) | 1.5(1.5,1.8) | 23(22.5,23) | 29(29,30.8) | 43(31.5,56) | 70(70,70) | 5(5,5) | 26(26,26) | 36.5(36.5,36.5) | 50(49,51) | 0.01 | 0.1 |
| T4 | Experienced-fellows | 50.5(48.8,52.3) | 3.5(1.75,4) | 19(16.8,19.5) | 25(22.3,28.8) | 61(57.5,64.5) | 72.5(71.8,73.3) | 9(9,9) | 24.5(23.8,25.3) | 36.5(36.5,36.5) | 43(39.5,46.5) | 0.01 | 0.01 |
| T5 | Residents | None |  |  |  | None | 69(68.5,69.5) | 4.8(4.1,5.4) | 26(24.5,27.5) | 36.5(36.5,36.5) | 66(61,71) |  |  |
| T6 | Residents | None |  |  |  | None | 75.5(74.8,76.3) | 9(9,9) | 26.8(25.6,27.9) | 36.5(36.5,36.5) | 62.5(61.3,63.8) |  |  |
| T7 | Residents | None |  |  |  | None | 72.5(71.8,73.3) | 8.3(7.9,8.6) | 26(26,26) | 36.5(36.5,36.5) | 65(57.5,72.5) |  |  |

**Abbreviations:** HOST-CHS: Hands-On Surgical Training–Congenital Heart Surgery; VSD: Ventricular Septum Defect

_a,_ P value^*^: Total HOST-CHS score between pretest and posttest cases performed by trainees from group A

_b,_ P value^#^: VSD closure time between pretest and posttest cases performed by trainees from group A

**Table E4 Details of VSD Closure in the Pretest**

| **Patient NO** | **Age**  **(month)** | **Weight**  **(kg)** | **Male** | **Surgeon** | **VSD diameter(min)** | **TV chord division** | **Total Cross-clamp(min)** | **First cross-clamp(min)** | **Second cross-clamp(min)** | **Multi- pump Run** | **CPB duration(min)** | **Residual Lesions** |
| --- | --- | --- | --- | --- | --- | --- | --- | --- | --- | --- | --- | --- |
| 1 | 8 | 6.5 | No | T1 | 8 | Yes | 50 | 50 | N/A | No | 92 | No |
| 2 | 10 | 6.6 | Yes | T1 | 6 | No | 51 | 51 | N/A | No | 152 | No |
| 3 | 10 | 6.8 | Yes | T1 | 7 | No | 70 | 70 | N/A | No | 152 | No |
| 4 | 20 | 14.5 | Yes | T2 | 11 | No | 86 | 86 | N/A | No | 110 | No |
| 5 | 336 | 48 | No | T2 | 13 | No | 76 | 76 | N/A | No | 102 | No |
| 6 | 17 | 12 | Yes | T3 | 6 | Yes | 129 | 45 | 84 | Yes | 140 | **Major Patch leak Qp:Qs=**1.7 |
| 7 | 12 | 9.5 | Yes | T3 | 7 | No | 99 | 83 | 16 | Yes | 139 | **Major Patch leak Qp:Qs=**1.6 |
| 8 | 5 | 5 | Yes | T3 | 5 | No | 95 | 78 | 17 | Yes | 113 | **Major Patch leak Qp:Qs=**1.8 |
| 9 | 14 | 12 | No | T4 | 8 | No | 99 | 50.3 | 48.7 | Yes | 109 | **Major Patch leak Qp:Qs** =.6 |
| 10 | 7 | 7.1 | Yes | T4 | 10 | Yes | 70 | 70 | N/A | No | 148 | No |
| 11 | 14 | 12 | No | T4 | 11 | No | 90 | 90 | N/A | No | 109 | No |

**Abbreviations:** VSD: Ventricular Septum Defect; CPB: Cardiopulmonary Bypass

**Table E5 Details of VSD Closure in the Posttest**

| **Patient NO** | **Age**  **(month)** | **Weight**  **(kg)** | **Male** | **Surgeon** | **VSD diameter(mm)** | **TV chord division** | **Total Cross-clamp(min)** | **Multi-pump Run** | **CPB duration(min)** | **Residual lesions** |
| --- | --- | --- | --- | --- | --- | --- | --- | --- | --- | --- |
| 12 | 10 | 9 | Yes | T1 | 10 | No | 53 | No | 72 | No |
| 13 | 6 | 6 | Yes | T1 | 5 | No | 53 | No | 97 | No |
| 14 | 3 | 4.2 | No | T1 | 6 | No | 45 | No | 73 | No |
| 15 | 36 | 16 | Yes | T2 | 8 | Yes | 64 | No | 81 | Minor patch leak(Qp:Qs=1.1) |
| 16 | 60 | 16 | No | T2 | 10 | No | 70 | No | 99 | No |
| 17 | 11 | 9 | No | T3 | 8 | No | 60 | No | 69 | No |
| 18 | 4 | 6.5 | No | T3 | 5 | No | 67 | No | 82 | No |
| 19 | 40 | 20 | Yes | T4 | 9 | No | 43 | No | 79 | No |
| 20 | 16 | 12 | Yes | T4 | 7 | No | 62 | No | 99 | No |
| 21 | 9 | 6.5 | No | T5 | 7 | No | 90 | No | 112 | No |
| 22 | 14 | 14 | Yes | T5 | 10 | No | 70 | No | 89 | No |
| 23 | 15 | 13 | No | T6 | 8 | No | 76 | No | 98 | No |
| 24 | 11 | 13 | Yes | T6 | 11 | Yes | 77 | No | 101 | No |
| 25 | 8 | 7.3 | Yes | T7 | 7 | No | 64 | No | 78 | No |
| 26 | 10 | 6.5 | No | T7 | 9 | No | 97 | No | 109 | No |

**Abbreviations:** VSD: Ventricular Septum Defect; CPB: Cardiopulmonary Bypas

**Table E6 Survey on the Trainees after Simulation.**

|  | **1 Strongly disagree** | **2 Disagree** | **3 Neither agree or disagree** | **4 agree** | **5 Strongly agree** |
| --- | --- | --- | --- | --- | --- |
| **Do you agree the simulation improves your surgical skill of VSD closure?** |  |  |  | **2/7（28.6%）** | **5/7（71.4%）** |
| **Do you agree the simulation improves VSD patch preparation?** |  |  | **1/7（14.3%）** | **1/7（14.3%）** | **5/7（71.4%）** |
| **Do you agree the simulation improves suturing of VSD patch?** |  |  | **2/7（28.6%）** | **1/7（14.3%）** | **4/7（57.1%）** |
| **Do you agree the simulation improves patch assessment?** |  |  | **1/7（14.3%）** |  | **6/7（85.7%）** |
| **Do you agree the simulation helps you to avoid collateral damage?** |  |  |  | **1/7（14.3%）** | **6/7（85.7%）** |
